# Prognostic Prediction Model for Glioblastoma: A Ferroptosis-Related Gene Prediction Model and Independent External Validation

**DOI:** 10.3390/jcm12041341

**Published:** 2023-02-08

**Authors:** Wenlin Chen, Chuxiang Lei, Yuekun Wang, Dan Guo, Sumei Zhang, Xiaoxi Wang, Zixin Zhang, Yu Wang, Wenbin Ma

**Affiliations:** 1 Department of Neurosurgery, Peking Union Medical College Hospital, Chinese Academy of Medical Sciences and Peking Union Medical College, Beijing 100730, China; 2 Department of Cardiac Surgery, State Key Laboratory of Cardiovascular Diseases, National Center for Cardiovascular Diseases, Fuwai Hospital, Chinese Academy of Medical Sciences and Peking Union Medical College, Beijing 100037, China; 3 Clinical Biobank, Medical Research Center, Peking Union Medical College Hospital, Chinese Academy of Medical Sciences and Peking Union Medical College, Beijing 100730, China

**Keywords:** differently expressed genes, ferroptosis, glioblastoma, prognosis, risk score

## Abstract

Glioblastoma (GBM) is the most common primary malignant intracranial tumor with a poor prognosis. Ferroptosis is a newly discovered, iron-dependent, regulated cell death, and recent studies suggest its close correlation to GBM. The transcriptome and clinical data were obtained for patients diagnosed with GBM from TCGA, GEO, and CGGA. Ferroptosis-related genes were identified, and a risk score model was constructed using Lasso regression analyses. Survival was evaluated by univariate or multivariate Cox regressions and Kaplan–Meier analyses, and further analyses were performed between the high- and low-risk groups. There were 45 ferroptosis-related different expressed genes between GBM and normal brain tissues. The prognostic risk score model was based on four favorable genes, *CRYAB, ZEB1, ATP5MC3,* and *NCOA4*, and four unfavorable genes, *ALOX5*, *CHAC1, STEAP3,* and *MT1G*. A significant difference in OS between high- and low-risk groups was observed in both the training cohort (*p* < 0.001) and the validation cohorts (*p* = 0.029 and 0.037). Enrichment analysis of pathways and immune cells and functioning was conducted between the two risk groups. A novel prognostic model for GBM patients was developed based on eight ferroptosis-related genes, suggesting a potential prediction effect of the risk score model in GBM.

## 1. Introduction

Glioblastoma (GBM) is the most common primary malignant tumor of the central nervous system (CNS), with an average annual incidence of 3.44 per 100,000 [1]. Among all the CNS primary tumors, GBM is classified as World Health Organization (WHO) grade 4 and shows the highest malignancy and the most invasiveness [2,3]. In 2010, the TCGA research team further classified GBM into classical, neural, mesenchymal, and proneural subtypes based on transcriptome expression data [4]. In recent years, with the development of exploring mutational profiling, gene expression, DNA methylation, and the tumor immune microenvironment, we have explored the molecular subtypes of GBM in greater depth and propose an integrated treatment scheme based on the novel molecular subtypes [5]. Current clinical treatment options for GBM include surgery, radiotherapy, chemotherapy, immunotherapy, targeted therapy, and tumor-treating fields [6,7], but the prognosis remains unfavorable. The Chinese Glioma Collaboration Group (CGCG) reported that the median overall survival (OS) of Chinese GBM patients was only 14.4 months in 2016 [8]. Meanwhile, the Central Brain Tumor Registry of the United States (CBTRUS) reported the mOS was eight months for American GBM patients regardless of treatment, while the five-year survival rate was only 7.2% [1]. Considering the poor prognosis status and the vacancy of widely accepted prognostic predictive models, the construction of a prognostic prediction model is of great significance.

Ferroptosis is a newly discovered, unique, iron-dependent form of nonapoptotic cell death triggered by the small molecule erastin, and it is independent of apoptosis, pyrolysis, and autophagy, although the current understanding of this pathway is not complete. The first report on ferroptosis, in which the researchers discovered that ferroptosis could be triggered by erastin and differed from apoptosis morphologically, biochemically, and genetically, was published in 2012 [9]. Further, activation of ferroptosis results in the nonapoptotic destruction of certain cancer cells, whereas inhibition of this process may protect organisms from neurodegeneration. Further research indicated the critical regulatory value of system Xc- and glutathione peroxidase 4 (*GPX4*) in the initiation and regulation of the ferroptosis process [10]. P53 was recognized as a ferroptosis sensitizer by inhibiting cystine uptake [11], while *FSP1* was explored as a protective factor against ferroptosis mediated by ubiquinone [12]. The role of ferroptosis in cancer remains a hotspot of research, and a current study suggests ferroptosis showed a double-edged sword role [13]. Immune cells were also involved in the regulation of ferroptosis in tumor cells, and CD8+ T cells functioned in enhancing ferroptosis-specific lipid peroxidation [14,15]. Several studies have explored the mechanism of ferroptosis in the genesis and development of GBM. The *Nrf2-Keap1* pathway was found to be an upstream agent regulating ferroptosis by affecting the expression of system Xc- in GBM cells [16], and *RSL3* and *APOC1* were proven to be driver alterations of the biological process [17,18]. Neutrophils could function as a ferroptosis inducer in GBM mouse models [19]. A potential pro-tumorigenic role of ferroptosis and a correlation between ferroptosis and GBM necrosis was also discovered [19].

Due to the current research findings in ferroptosis, the expression levels of ferroptosis-related genes may act as prognostic factors in GBM. In this study, we identified the ferroptosis-related prognostic differently expressed genes (DEGs) between GBM and normal brain tissues through systematic database retrieval, as well as the corresponding clinical information. The prognostic prediction model was further constructed and validated. We also explored the difference in function and the tumor immune microenvironment. Our findings suggest that ferroptosis-related genes are vital in predicting GBM patients’ prognosis and indicate potential novel tumor markers or therapeutic targets for GBM treatment.

## 2. Materials and Methods

### 2.1. Study Design and Data Acquisition

The transcriptome profiles and clinical datasets of GBM patients were collected from The Cancer Genome Atlas (TCGA), the Chinese Glioma Genome Atlas (CGGA), and the Gene Expression Omnibus (GEO) (microarray data of GSE83300 [20], GSE74187 [21], and GSE13041 [22]). For the TCGA data, we used organized mRNAseq data by screening the transcriptome data of GBM patients. For the CGGA data, we used the organized mRNAseq data, mRNAseq_693 and mRNAseq_325 (Illumina Hiseq). The transcriptome sequencing data of normal brain tissue originated from the Genotype-Tissue Expression (GTEx) database as a normal control. Profiles from the TCGA database comprised the training cohort, and the validation cohort consisted of patients’ information from the CGGA and GEO databases, separately. OS was our evaluation criterion for patients’ survival, and progression-free survival (PFS) was not an evaluation criterion due to the incomplete data recording of the database. The included series were from histologically diagnosed human GBM tissue that had detailed transcriptome and clinical profiles, including the exact prognostic status and overall survival. Conversely, data collected from GBM cell lines without intact transcriptome and clinical profiles contained fewer than 30 samples or had a follow-up time of fewer than 30 days and were excluded. Surrogate variable analysis (SVA) was implemented to normalize batch effects between different chips using the “sva” R package.

### 2.2. Identification of DEGs and Ferroptosis-Related DEGs

DEGs between GBM (the TCGA training cohort) and normal brain tissues (the GTEx normal controls) were selected using the “limma” package (version 3.44.3) in R (version 4.0.2), and the threshold for significant differential expression was determined as an absolute log2-fold change (FC) > 2 and a false discovery rate (FDR) < 0.05. The fold change (FC) represents the ratio of expression between two groups, and the false discovery rate (FDR) is obtained by correcting for the *p*-value of the significance of differences. The list of ferroptosis-related genes was obtained from the Human Gene Database (https://www.genecards.org) (accessed on 17 July 2021), and 60 genes were obtained as ferroptosis-related genes (Supplementary Table S1). Ferroptosis-related DEGs between GBM and normal brain tissues were likewise realized by the “limma” R package. Due to the small number of included genes, the criteria for statistical significance were defined as an absolute logFC > 0.3 and an FDR < 0.05.

### 2.3. Selection of Ferroptosis-Related Prognostic DEGs

Univariate Cox regression analyses of all ferroptosis-related genes were conducted to filter out genes associated with patients’ survival, and *p* < 0.05 was defined as statistically significant. The ferroptosis-related genes associated with patients’ survival rates were defined as ferroptosis-related prognostic genes. Next, we took an intersection of ferroptosis-related DEGs and ferroptosis-related prognostic genes, and the results obtained from the intersection were ferroptosis-related prognostic DEGs.

### 2.4. Establishment of the Predictive Model of Ferroptosis-Related Prognostic DEGs

Lasso-penalized Cox regression analyses were performed of those ferroptosis-related prognostic DEGs obtained in the previous analysis to identify the contributing genes and construct the prognostic risk score model [23]. The prognostic risk score was determined by the summation of each chosen gene’s expression multiplied by the corresponding coefficient, and the formula is shown as follows.

$$risk\;score=\sum_{i=1}^{n}{Coefficient}_{{mRNA}_{i}}\cdot expression\;of\;{mRNA}_{i}$$

The prognostic risk score of each patient was calculated and recorded. According to the median risk score of the training cohort, patients with GBM were then divided into high-risk and low-risk groups. Kaplan–Meier and time-dependent receptor operating characteristic (ROC) analyses were conducted to evaluate the differences and predictive ability of OS between high-risk and low-risk groups with the “survival”, “surviving” and “timeROC” packages in R. Principal component analyses (PCA) and t-distributed stochastic neighbor embedding (t-SNE) analyses were performed to reduce feature dimensionality and examine the degree of differentiation of the risk score model between the groups separately. Univariate and multivariate Cox regression analyses were enforced to verify whether the risk score model was independent of other OS prediction clinical features.

### 2.5. Validation of the Predictive Prognostic Model

The prognostic risk score model was validated in the CGGA and GEO datasets. Patients in both validation cohorts were separated into two risk groups according to the same standard of value as the training cohort. Analyses similar to the training group were conducted to validate the predictive ability and accuracy of the risk score model.

### 2.6. Functional and Immune Enrichment Analysis

Gene ontology (GO) and the Kyoto Encyclopedia of Genes and Genomes (KEGG) were conducted to investigate the function and pathways enrichment status of DEGs between the GBM and normal tissue and between the risk groups. The “ClusterProfiler” package of R was applied to visualize the analysis results.

Differences in immune status were explored between the high-risk and low-risk groups in both cohorts. A single sample gene set enrichment analysis (ssGSEA) was performed to describe the immune enrichment score of immune cell infiltration and the related function activation. The “GSVA” package of R was used in the implementation of the analysis.

### 2.7. Validation of Key Genes by the Quantitative Real-Time Polymerase Chain Reaction

We performed quantitative real-time polymerase chain reaction (qRT-PCR) on key genes to further validate the reliability of the data analysis and model-building process. GBM tissues and corresponding peritumoral tissues were obtained from six patients who underwent surgery. Total RNA was extracted from fresh frozen tissues using TRIzol (Invitrogen, Carlsbad, CA, USA) and reverse transcribed using a FastQuant RT kit (TIANGEN, Beijing, China) for reverse transcription. The qRT-PCR experiments were carried out using the CFX Connect™ Real-Time PCR Detection System (Bio-Rad, Berkeley, CA, USA) and Cat. No. AB1158B reagent (Invitrogen, Carlsbad, CA, USA), and glyceraldehyde-3-phosphate dehydrogenase (GAPDH) was used as an internal reference. The corresponding qRT-PCR primer sequences for each key gene are shown in Supplementary Table S2.

### 2.8. Statistical Analysis

Student’s *t*-tests, Wilcoxon tests, or Mann–Whitney *U* tests were performed as appropriate methods for measuring differences in scale or ordinal variables. Survival analyses were conducted with Cox proportional hazards regressions and Kaplan–Meier analyses. Two-sided *p* < 0.05 was considered to be statistically significant. All the statistical analyses were performed with R software v4.0.2 (R Foundation for Statistical Computing, Vienna, Austria).

## 3. Results

### 3.1. Identification of DEGs between GBM and Normal Brain Tissue

**Figure 1:**
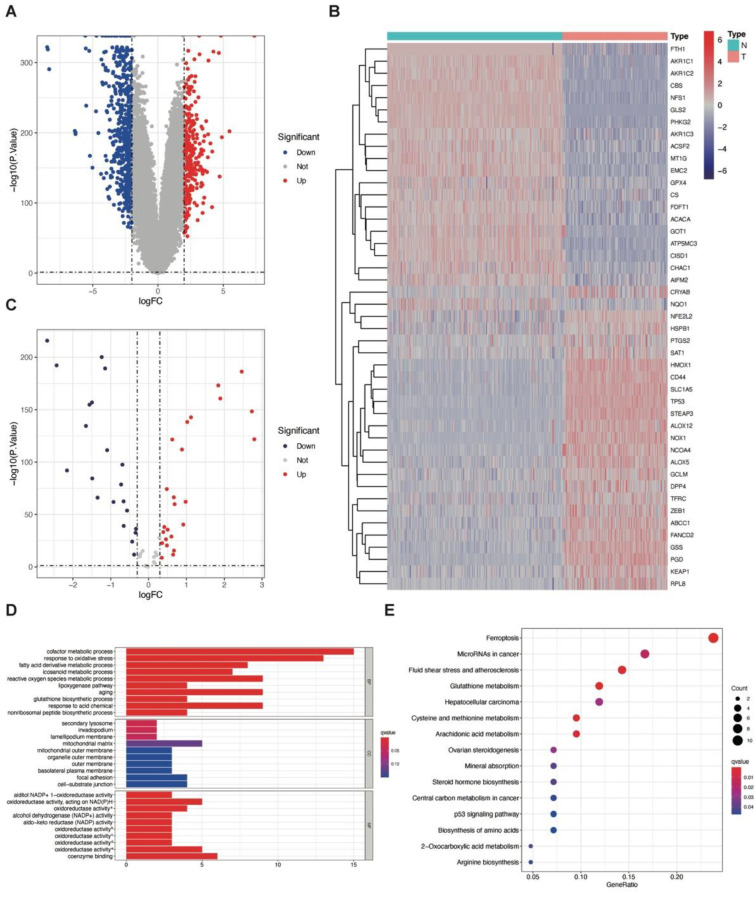
An overview of comprehensive and ferroptosis-related differentially expressed genes. (**A**) Volcano plot of all differentially expressed genes (DEGs) between GBM and normal brain tissue. (**B**) A heatmap of 45 ferroptosis-related genes showed the different expression patterns between GBM and normal brain tissue. (**C**) Volcano plot of 45 ferroptosis-related DEGs between GBM and normal brain tissue; 24 DEGs were upregulated and 21 DEGs were downregulated. (**D**) Gene set enrichment analysis of gene ontology (GO) among the ferroptosis-related DEGs. BP: biological process; CC: cellular component; MF: molecular function. (**E**) Gene set enrichment analysis of Kyoto Encyclopedia of Genes and Genomes (KEGG) among the ferroptosis-related DEGs. Gene ratio: the proportion of the enriched genes to the total amount of genes in a specific pathway.

A flow chart was developed to illustrate our study (Supplementary Figure S1). Populations of 159 GBM patients from the TCGA (training cohort), 374 GBM patients from the CGGA, 369 patients from the GEO (validation cohort) databases, and 290 normal brain samples from the GTEx database with transcriptome data were included for DEGs analyses. As the TCGA and GEO datasets did not clearly indicate the IDH mutation status, we included both IDH wildtype and mutant patients. Altogether, 870 genes were discovered to have significant differences in expression levels between GBM (the TCGA training cohort) and normal brain tissue (Figure 1A and Supplementary Table S3).

### 3.2. Identification and Gene Set Enrichment Analysis of Ferroptosis-Related DEGs between GBM and Normal Brain Tissue

According to previous reports, 60 genes were identified as ferroptosis-related genes in terms of ferroptosis. We identified 45 ferroptosis-related DEGs between the TCGA training cohort and GTEx normal control, in which 24 were upregulated and 21 were downregulated (Figure 1B,C; Supplementary Table S4).

To learn more about the pathways and function of ferroptosis-related DEGs between the GBM and normal brain, we conducted a gene set enrichment analysis of the GO and KEGG pathway analyses (Figure 1D,E). In the GO analysis, genes were enriched in the cofactor metabolic process and the oxidative stress response. In addition to ferroptosis, genes were also enriched in microRNAs in cancer in the KEGG analysis.

### 3.3. Lasso Regression Model and Kaplan–Meier Analysis of the Training and Validation Cohorts

**Figure 2:**
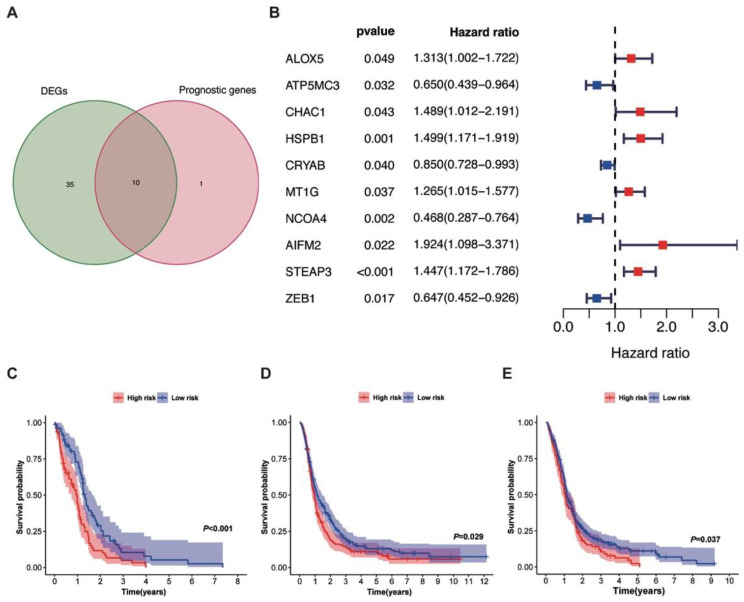
Ferroptosis-related prognostic DEGs and risk stratification based on them. (**A**) Venn diagram of ferroptosis-related DEGs and prognostic genes. (**B**) Univariate Cox regression analyses and forest plot of correlations between ferroptosis-related prognostic DEGs and GBM patients’ survival rates. (**C**–**E**) Kaplan–Meier curve of difference in survival showed patients in the low-risk group had a statistically significantly better prognosis than patients in the high-risk group in both the training cohort (TCGA) (**C**) and validation cohorts (CGGA (**D**) and GEO (**E**)). Red: high-risk group; blue: low-risk group; blue and red square: 95% confidence intervals (CIs).

To discover the ferroptosis-related prognostic genes, we conducted univariate Cox regression analyses and screened out 11 prognostic genes related to OS out of 60 ferroptosis-related genes. Taken at an intersection of the 45 ferroptosis-related DEGs and the 11 ferroptosis-related prognostic genes, ten genes were identified as ferroptosis-related prognostic DEGs and prepared as candidate gene sets for risk score prognostic model establishment (Figure 2A). Meanwhile, 35 ferroptosis-related DEGs showed no prognostic value, and one ferroptosis-related prognostic gene did not show a difference in the expression level between the GBM and normal tissues. Among the ferroptosis-related prognostic DEGs, four favorable genes related to better prognosis with hazard ratios (HRs) < 1 and six unfavorable genes prone to worse outcomes with HRs > 1 were detected by univariate cox regression analyses (Figure 2B).

The Lasso regression model was applied for the prognostic model establishment for OS in GBM patients based on ten ferroptosis-related prognostic DEGs. The model eventually identified eight genes for the model construction based on the optimal value of λ (Table 1). Among them, *CRYAB, ZEB1, ATP5MC3,* and *NCOA4* were favorable genes with negative coefficients and protective functions for GBM patients, and *ALOX5, CHAC1, STEAP3,* and *MT1G* were unfavorable genes with positive coefficients and declined OS rates.

The risk score was calculated for every patient based on the gene expression levels of the model construction genes and their corresponding coefficient, and the median risk score of the training cohort was calculated. The median risk score was used as a threshold dividing patients into high-risk (n = 79) and low-risk groups (n = 80) in the training cohort, and patients divided into the low-risk group showed an obviously longer OS rate than patients divided into the low-risk group (*p* < 0.001) using Kaplan–Meier analyses (Figure 2C).

The validation cohort (GEO and CGGA) was grouped according to the same median risk threshold from the training cohort after the model was established. Patients in the low-risk group also showed a statistically significantly better prognosis than patients in the high-risk group in both the CGGA (high-risk group = 168, low-risk group = 206, *p* = 0.029, Figure 2D) and the GEO (high-risk group = 111, low-risk group = 258, *p* = 0.037, Figure 2E) datasets.

### 3.4. ROC Analysis, PCA, and t-SNE Analyses of the Training and Validation Cohorts

**Figure 3:**
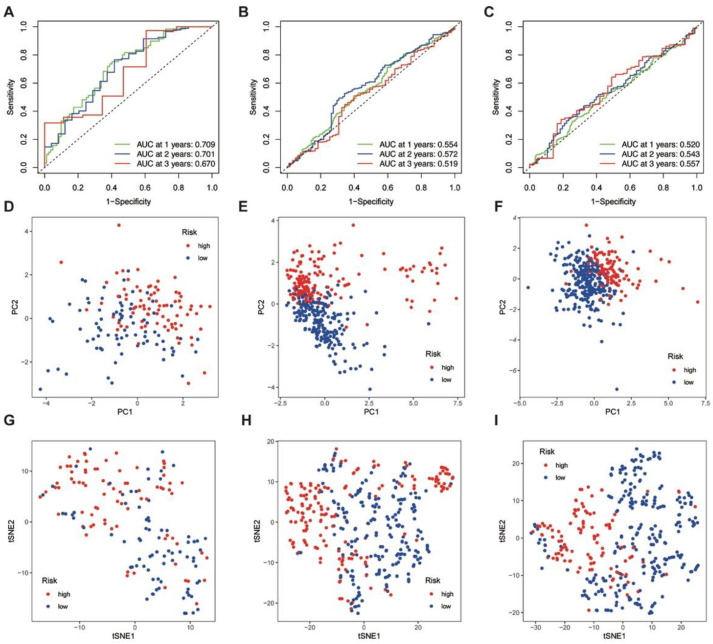
ROC analysis, PCA, and t-SNE analysis for risk score classification in GBM patients. (**A**–**C**) ROC analyses of risk scores for 1-, 2- and 3-year survival of GBM patients were conducted in TCGA (**A**), CGGA (**B**), and GEO (**C**). (**D**–**F**) The PCA supported the classification of GBM patients into high-risk and low-risk groups based on risk scores, respectively, in TCGA (**D**), CGGA (**E**), and GEO (**F**). (**G**–**I**) t-SNE analyses supported the stratification of GBM patients from TCGA (**G**), CGGA (**H**), and GEO (**I**) into high-risk and low-risk groups based on risk scores. ROC: receiver operating characteristic; PCA: principal component analysis; t-SNE: t-distributed stochastic neighbor embedding.

To further validate the effectiveness of the prognostic model, time-dependent ROC analyses were conducted in both the training and validation groups. The area under the curve (AUC) values for the risk score were 0.71 at 1 year, 0.70 at 2 years, and 0.67 at 3 years, suggesting a fair level of accuracy for OS prediction at 1 year and 2 years (Figure 3A). Meanwhile, the prognostic effect was not evident in the CGGA (Figure 3B) and GEO (Figure 3C) validation cohorts.

PCA and t-SNE analyses were conducted to reduce feature dimensionality and examine the model’s ability to distinguish between high- and low-risk groups. In the PCA plots, the two groups could be separated into two clusters in both the training and validation groups, indicating a satisfactory verifying capability (Figure 3D–F). The t-SNE analysis also showed discrete direction distribution and suggested consistency within two risk groups (Figure 3G–I).

### 3.5. Multivariate Cox Regression Analysis and Gene Set Enrichment Analysis of DEGs between Risk Groups

We included clinical information, such as age, gender, medical history, and IDH mutation status, to explore the comprehensive effect of multiple factors on the prognosis of patients with GBM.

**Figure 4:**
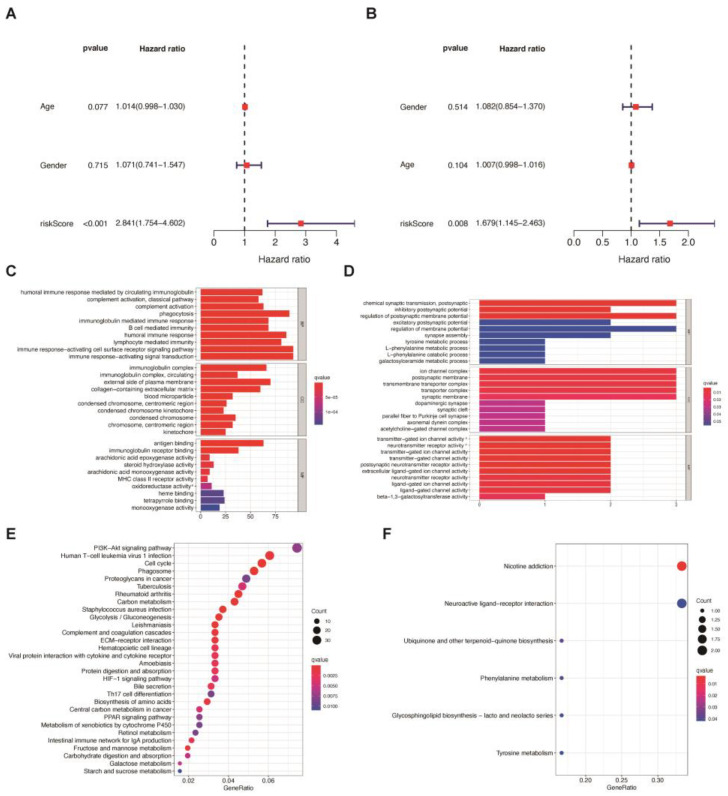
Multivariate Cox regression analysis for risk score classification and gene set enrichment analysis for DEGs between high-risk and low-risk groups. Red square: hazard ratio; blue line: 95% CIs. (**A**,**B**) Multivariate Cox regression analysis and forest plot of the survival in TCGA (**A**) and CGGA (**B**). (**C**,**D**) GO analysis among the DEGs between high-risk and low-risk groups in TCGA (**C**) and CGGA (**D**). (**E**,**F**). KEGG analysis among the DEGs between high-risk and low-risk groups in TCGA (**E**) and CGGA (**F**).

In the training cohort, age, gender, and risk score were included in the multivariate Cox regression analysis, as IDH mutation and MGMT promoter methylation status were not indicated in the TCGA database. Only risk score was recognized as a prognosis-related factor, with an HR of 2.841, while patients’ age and gender were not considered independent risk factors (Figure 4a). A similar result was also observed in the validation cohort (Figure 4b). The risk score was significantly related to prognosis with an HR of 1.679, and age and gender showed no significance in prognosis prediction. In addition, we further compared the effect of the risk score with molecular features and therapy options in the CGGA cohort. Multivariate Cox regression analyses suggested radiotherapy history, chemotherapy history, and IDH mutation as protective prognosis-related factors, with HRs of 0.691, 0.441, and 0.690, while risk score did not show equal predictive efficiency (Supplementary Figure S2).

To further explore the differences between risk groups, we conducted gene set enrichment analyses of the GO and KEGG pathway analyses on DEGs between the high-risk and low-risk groups in the training and validation cohorts. Using limma with an absolute logFC > 2 and an FDR < 0.05, we identified 1180, 51, and 187 DEGs between risk groups in the TCGA training cohort, the CGGA, and the GEO validation cohort, respectively (Supplementary Tables S5–S7). In the GO analysis, DEGs were enriched in phagocytosis, the immune response-activating cell surface receptor signaling pathway, immune response-activating signal transduction, and the humoral immune response in the training cohort (Figure 4C). In the CGGA validation cohort, chemical synaptic transmission (postsynaptic), regulation of postsynaptic membrane potential, the ion channel complex, the postsynaptic membrane, the transmembrane transporter complex, and the transporter complex were markedly enriched (Figure 4D), while translational pathways, such as co-translational protein targeting to the membrane and the establishment of protein localization to the membrane were obviously enriched in the GEO validation cohort (Supplementary Figure S3C). In the KEGG analysis, DEGs were enriched in the PI3K-Akt signaling pathway, human T-cell leukemia virus 1 infection, the cell cycle, and phagosome in cancer in the training cohort (Figure 4E) and nicotine addiction, coronavirus disease, the ribosome-related pathway, and the neurodegeneration pathway in the validation cohort (Figure 4F, Supplementary Figure S3B).

### 3.6. Immune Enrichment Score of High-Risk and Low-Risk Groups

**Figure 5:**
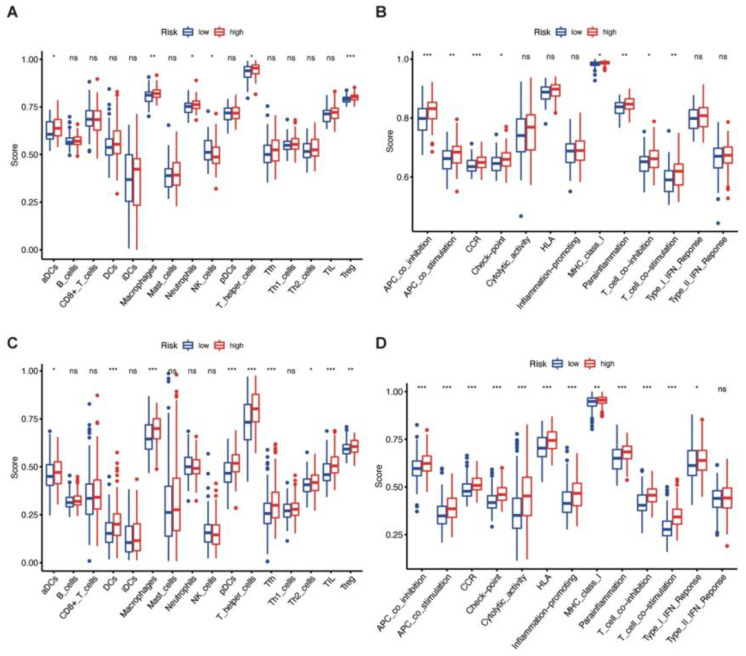
ssGSEA score of immune microenvironments and functions between high-risk and low-risk groups. (**A**,**C**) Composition of tumor-infiltrating immune cell types of high-risk and low-risk groups in TCGA (**A**) and CGGA (**C**). (**B**,**D**) Distribution of immune function and relevant pathways of high-risk and low-risk groups in TCGA (**B**) and CGGA (**D**). ssGSEA: single sample gene set enrichment analysis. Blue and red dot: outliner records; *: *p* < 0.05; **: *p* < 0.01; ***: *p* < 0.001.

To further investigate the differences in immune microenvironment and function between the two risk groups, ssGSEA was performed. We focused on immune cell infiltration and immunologically relevant functions and pathways. An obvious overlap was observed in immune cell infiltration between the training (Figure 5A) and validation cohorts (Figure 5C). The immune enrichment scores of aDCs, macrophages, T-helper cells, and T regulatory cells (Tregs) were significantly higher in high-risk groups in both cohorts. Neutrophils were upregulated and NK cells were downregulated significantly in the high-risk group of the training cohort, while levels of DCs, pDCs, follicular helper T cell (Tfh), T-helper 2 (Th2) cells, and tumor-infiltrating lymphocytes (TIL) varied in the validation cohort.

Several discrepancies in immune function also existed among the two risk groups. APC co-inhibition and co-stimulation, CCR, checkpoint, MHC class I, para-inflammation, and T cell co-inhibition and co-stimulation were markedly enriched in high-risk groups in both cohorts (Figure 5B,D). In contrast, cytolytic activity, HLA, inflammation-promoting, and type I IFN responses were significantly enriched in the training cohort.

### 3.7. Validation of the Key Genes and Related Proteins in Human Tissues

**Figure 6:**
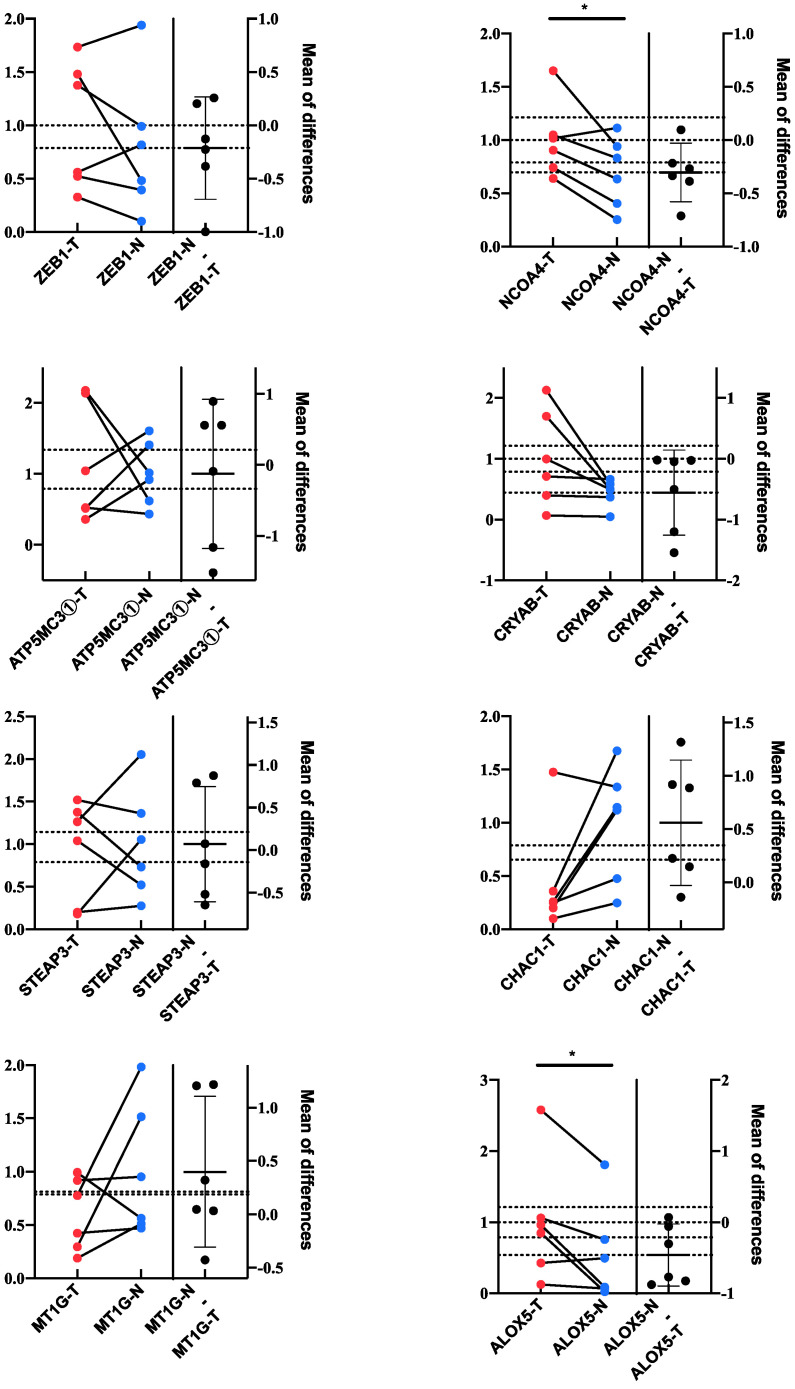
Validation of the crucial genes in the prognostic model. Quantitative real-time polymerase chain reaction (qRT-PCR) of the 8 key genes. *: *p* < 0.05.

To further validate the reliability of the bioinformatics analysis of the dataset, we performed qPCR in six GBM tissues and the corresponding peritumoral tissues of patients to determine the key genes in the prognostic model. At the transcriptional level, we found 2 genes, *NCOA4* and *ALOX5*, had significant differences in expression between GBM and normal brain tissues by qRT-PCR, while 4 genes, *CHAC1, CRYAB3, ZEB1,* and *STEAP3*, changed in the same direction as the public dataset, but did not reach statistical significance. In addition, the trends of changes in *ATP5MC3* and *MT1G* gene expression were not significant (Figure 6).

## 4. Discussion

Known as an iron-dependent programmed cell death, ferroptosis has attracted increasing attention in recent years, and its molecular mechanisms and correlated pathways have been further studied. Growing evidence has displayed the critical role of ferroptosis in tumorigenesis and its ability to become a potential treatment approach [24]. However, research on the influence of ferroptosis in the tumor biology of GBM is still rare, and enough concrete evidence has yet to be clarified on the role of ferroptosis-related genes. In this study, we built a ferroptosis prognostic risk score model for GBM patients based on high-throughput expression analysis. Eight ferroptosis-related prognostic DEGs were identified and applied to model establishment. Preliminary verifications of the predictive ability and accuracy were conducted by both internal and external validation. Moreover, function and immune enrichment analyses between high and low-risk groups indicated stratified and significant differences in OS between groups in both training and validation cohorts. However, the accuracy of the prediction model still needs to be improved.

Previous studies suggested that the Nrf2-Keap1 pathway plays an essential role in ferroptosis in GBM cells [16], while neutrophils were discovered to trigger ferroptosis in the GBM mouse model [19]. A few studies have focused on ferroptosis-related prognostic models in glioma patients. Liu et al. [25] and Zhuo et al. [26], respectively, conducted two ferroptosis-related gene signatures in glioma in 2020, in which *HSPB1, CISD1,* and *AKR1C2* overlapped in these two models, while a prognostic predictive model specifically for low-grade gliomas was constructed [27]. Compared with the previously published research, we focused only on GBM due to the significant differences in gene expression and prognosis of different glioma pathological features. High-throughput omics were applied, and a lasso-penalized Cox regression analysis was conducted, which is believed to be more accurate than a single gene prediction model or stepwise selection. We also explored the independent prognostic prediction value of this model, while the results of the analysis suggested the synergist role of this model and IDH mutation, radiotherapy, and chemotherapy. Further, clinical validation using tumor samples was conducted. These results indicated that the ferroptosis process in GBM cells was associated with GBM patients’ survival, thus suggesting the potential therapeutic target function for ferroptosis-related pathways and molecules.

A total of eight genes were included in the risk score prognostic model, including favorable genes, *CRYAB, ZEB1, ATP5MC3, and NCOA4*, and unfavorable genes, *ALOX5, CHAC1, STEAP3,* and *MT1G*. Most of these included genes played a vital role in tumor genesis and development according to previous studies. Meanwhile, compared to previously published studies, *ALOX5, NCOA4, and STEAP3* were overlapped. The elevation of the expression level of *NCOA4* was proven to result in the degradation of ferritin and an increase in the intracellular ferrous iron level, and ultimately, ferroptosis in laboratory research [28]. *ALOX5* and its coded protein, 5-LO, has long been recognized as a predictive marker for patients with GBM, which was consistent with the prediction model [29,30], while *STEAP3* was reported as a negative predictor of OS for patients with GBM [31]. Further research exploring the hidden connection among the prognostic ferroptosis-related genes is required. *CRYAB, ZEB1, ATP5MC3, CHAC1,* and *MT1G* were first proposed in our study. *CRYAB* functions as a molecular chaperone with anti-apoptotic activity and was significantly correlated to the tumorigenesis of GBM cells, while clinical research was vacant [32,33]. *CHAC1* functioned in the temozolomide response [34]. Notably, *ZEB1* was widely recognized as an accelerating factor for the genesis and invasion of GBM and was inversely correlated with survival [35,36]. In comparison, we found that *ZEB1* expression was protective in the risk score. Therefore, the exact roles of *ZEB1* in the ferroptosis-related process in GBM patients need further exploration.

PCA and t-SNE were conducted for dimensional reduction assessment. PCA is the most commonly used dimensional reduction assessment, which is unsupervised learning and has a fast analysis speed. However, there are relative features lost when high-dimensional data are transformed into low-dimensional data. t-SNE analysis has higher complexity and accuracy, so it is suitable for in-depth analysis. Meanwhile, function and immune enrichment annotation were performed to explore the difference between the two risk groups. Enrichment pathways include the *PI3K-Akt* signaling pathway, transmembrane transporter complex, ion channel complex, cell cycle, and the immune response-activating cell surface receptor signaling and signal transduction pathway. The protective effect of hyperactive mutation of the *PI3K-Akt-mTOR* signaling pathway was observed by decreasing ferroptosis [37]. Glutamine transporter *SLC1A5* was also proved to be a target for ferroptosis regulation in melanoma [38]. Recent studies indicated that cell cycle arrest and ferroptosis could be induced by dihydroartemisinin [39]. The immune cell enrichment also suggested an increase in DCs, macrophages, and neutrophils in high-risk groups. The function of neutrophils in ferroptosis was a research hotspot, and several studies put forward the ferroptosis-accelerating effect of neutrophils [19]. Macrophages were discovered to function in the ferroptosis process in pancreatic tumor cells [40]. Several enriched immune pathways include checkpoint, MHC class I, para-inflammation, T cell co-inhibition and co-stimulation, cytolytic activity, and type I IFN response. Though the function of some immune cells or pathways in ferroptosis has been studied, further exploration should be accomplished for potential treatment targets.

There are several limitations to our study. First, although the separation effect was sufficient, the accuracy of our prognostic predictive model needs further improvement in the validation cohorts. This drawback might be caused by the inconsistency of the demographic baseline information between the training and the validation set and could be enhanced with more ferroptosis-related genes and more cases included during the growing understanding of ferroptosis. Second, Lasso regression makes some coefficients smaller, and even some coefficients with smaller absolute values become zero directly, therefore excluding some prognostic significant genes from the predictive model. Further, current studies have proposed that GBM patients with different molecular subtypes have differences in gene expression, methylation profiles, prognosis, and treatment response. However, our study could not distinguish the molecular subtypes of the enrolled patients due to the limitations of raw data, which may introduce bias. Further, some genes were located in the same segment of the chromosome, making it difficult to rule out chromosomal physical factors. Furthermore, more well-designed basic research is essential to reveal the mechanism of genes, pathways, and immune cells with ferroptosis in the tumor biology and treatment regimens of GBM.

## 5. Conclusions

In conclusion, our study put forward a novel ferroptosis-related prognostic predictive model for GBM based on the TCGA dataset and validated by CCGA and GEO datasets and clinical samples. With respect to the present findings, our model is able to calculate the risk score according to the expression of specific ferroptosis-related genes to a certain extent and predict the survival status of GBM patients. The results also suggested alterations in the metabolic microenvironment and indicated potential directions for identifying biomarkers for diagnosis and treatment.

## Figures and Tables

**Table 1:** Genes with Log2FC, FDR, and their coefficients in the prognostic model after LASSO regression.

Gene Symbol	Coefficient ^†^	Training Cohort		Validation Cohort		Chromosome Localization
		Log_2_FC ^‡^	FDR ^¶^	Log_2_FC ^‡^	FDR ^¶^	
*ALOX5*	0.13724394	1.167	<0.001 *	0.076	NS	10q11.21
*CHAC1*	0.13378105	0.250	NS	0.085	NS	15q15.1
*STEAP3*	0.11443311	1.005	<0.001 *	0.158	NS	2q14.2
*MT1G*	0.08304952	0.217	NS	−0.007	NS	16q13
*CRYAB*	−0.0297374	2.106	<0.001 *	−0.059	NS	11q23.1
*ZEB1*	−0.2148601	−0.143	NS	−0.119	NS	10p11.22
*ATP5MC3*	−0.4763973	−0.136	NS	−0.075	NS	2q31.1
*NCOA4*	−0.5506693	−0.097	NS	−0.107	NS	10q11.22

^‡^ Log_2_FC = Log_2_(mean expression of high-risk group/mean expression of low-risk group). ^¶^ FDR was calculated by *p*-value from the Wilcoxon test. ^†^ Coefficients were calculated by Lasso regression. * marks significant differences. Log_2_FC: Log_2_ fold change; FDR: false discovery rate. NS: not significant.

## Data Availability

The clinical and transcriptome data were collected from the TCGA (https://portal.gdc.cancer.gov) (accessed on 17 July 2021), CGGA (http://www.cgga.org.cn) (accessed on 17 July 2021), GEO (https://www.ncbi.nlm.nih.gov/gds) (accessed on 17 July 2021), and GTEx (https://gtexportal.org/home) (accessed on 17 July 2021) public databases.

## Supplementary Materials

The following supporting information can be downloaded at: https://www.mdpi.com/article/10.3390/jcm12041341/s1, Figure S1. Flow chart of the study. Figure S2. Multivariate Cox regression analysis and forest plot of the survival in CGGA (including radiotherapy, chemotherapy, and IDH status). Figure S3. DEGs in GEO dataset and gene set enrichment analysis for DEGs between high-risk and low-risk groups. A. Volcano plot of DEGs between high- and low-risk groups in the GEO dataset. B. Gene set enrichment analysis of KEGG among the ferroptosis-related DEGs. C. Gene set enrichment analysis of GO among the ferroptosis-related DEGs. Table S1. The list of 60 ferroptosis-related genes. Table S2. Metabolic key genes corresponding to qRT-PCR primer sequences. Table S3. The list of 870 differentially expressed genes. Table S4. The list of 45 ferroptosis-related DEGs between the TCGA training cohort and the GTEx normal control. Table S5. The list of DEGs between risk groups in the TCGA training cohort. Table S6. The list of DEGs between risk groups in the CGGA validation cohort. Table S7. The list of DEGs between risk groups in the GEO validation cohort.
