# Supplementary figures and images for: Prognostic Prediction Model for Glioblastoma: A Ferroptosis-Related Gene Prediction Model and Independent External Validation
Source: J Clin Med. 2023 Feb 8;12(4):1341. doi: 10.3390/jcm12041341 (PMC9960289; doi:10.3390/jcm12041341)

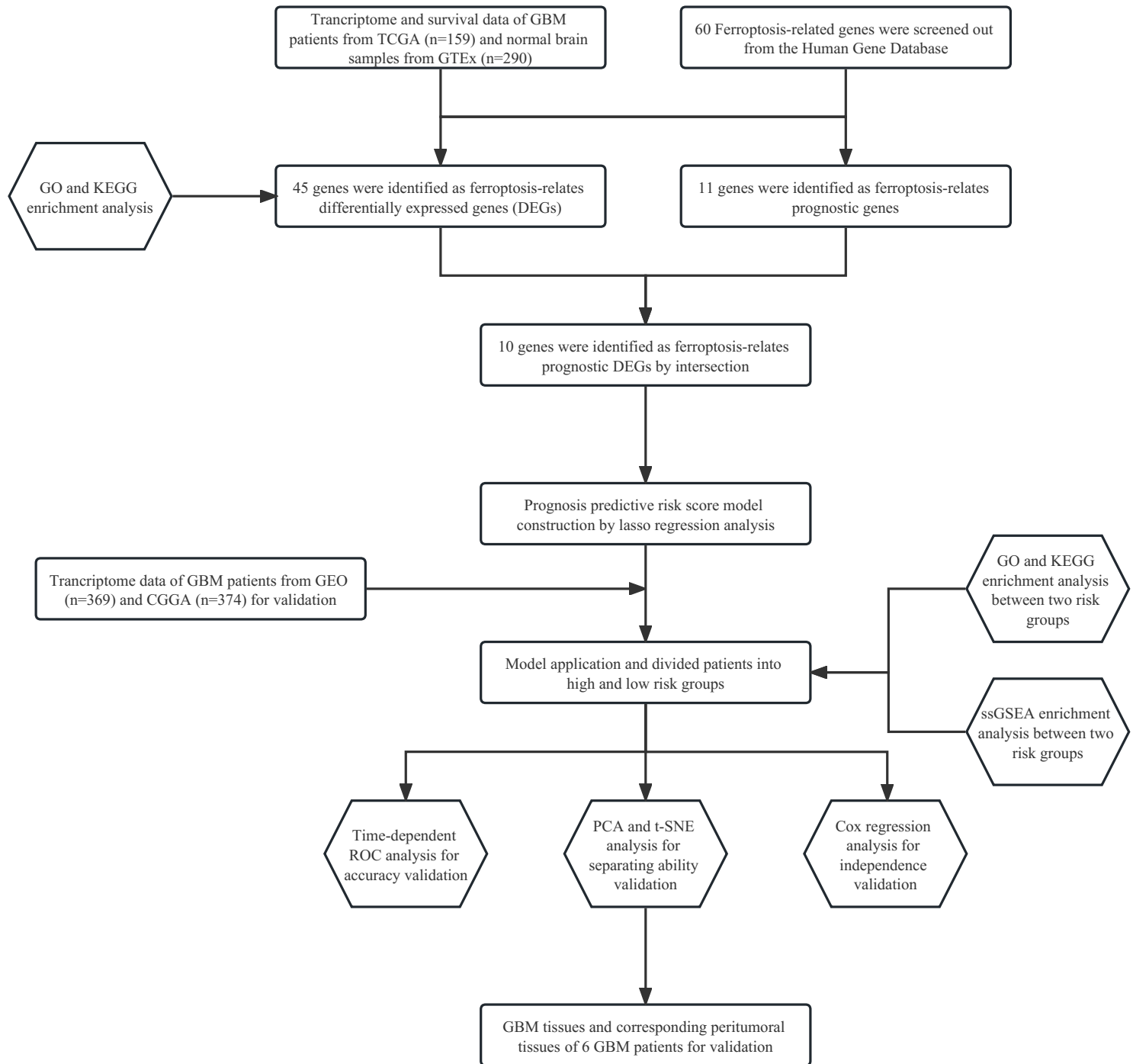

Supplement: Supplementary file 1 [file jcm-12-01341-s001.zip › Supplementary Figure S1.pdf]

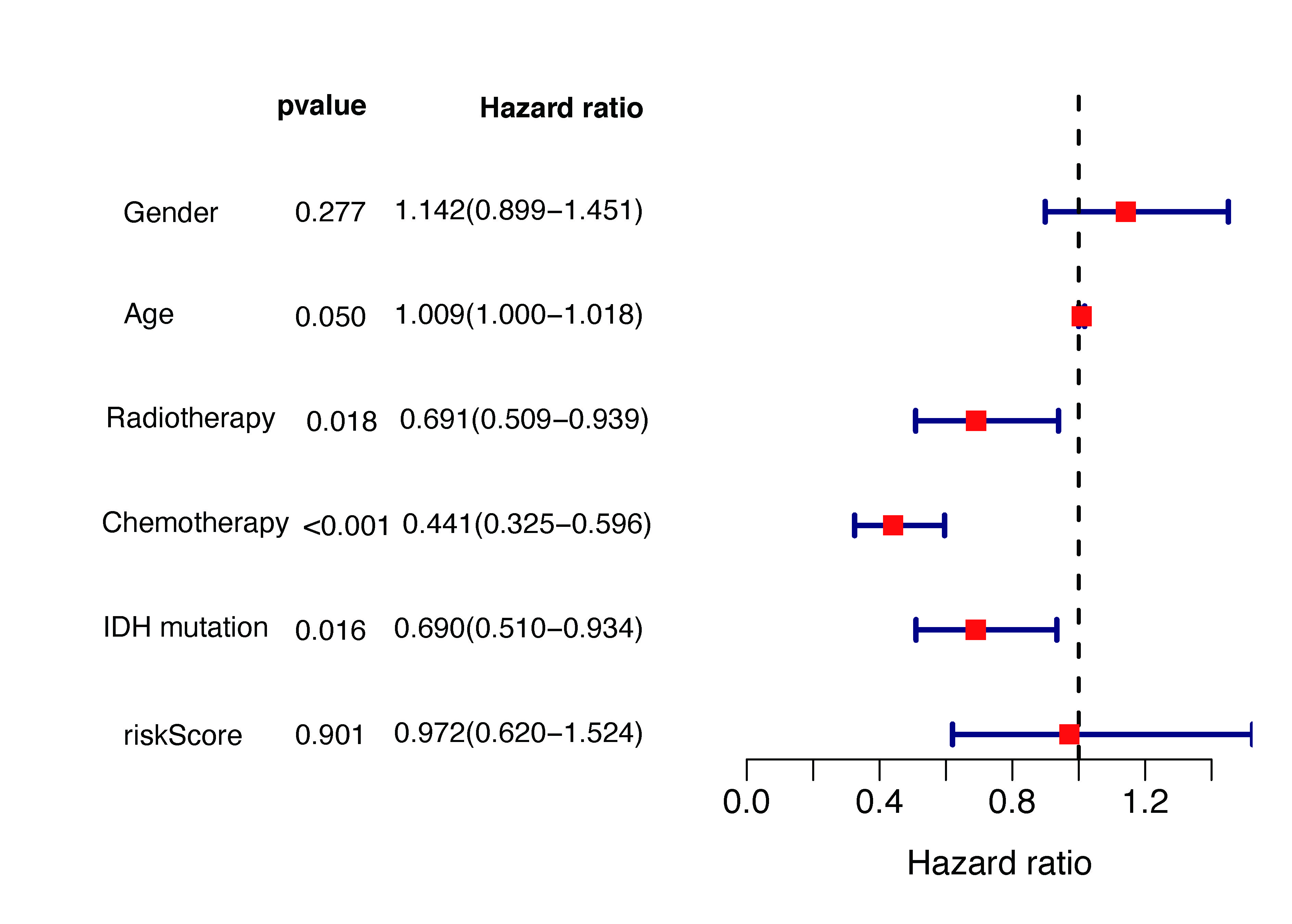

Supplement: Supplementary file 1 [file jcm-12-01341-s001.zip › Supplementary Figure S2.tif]

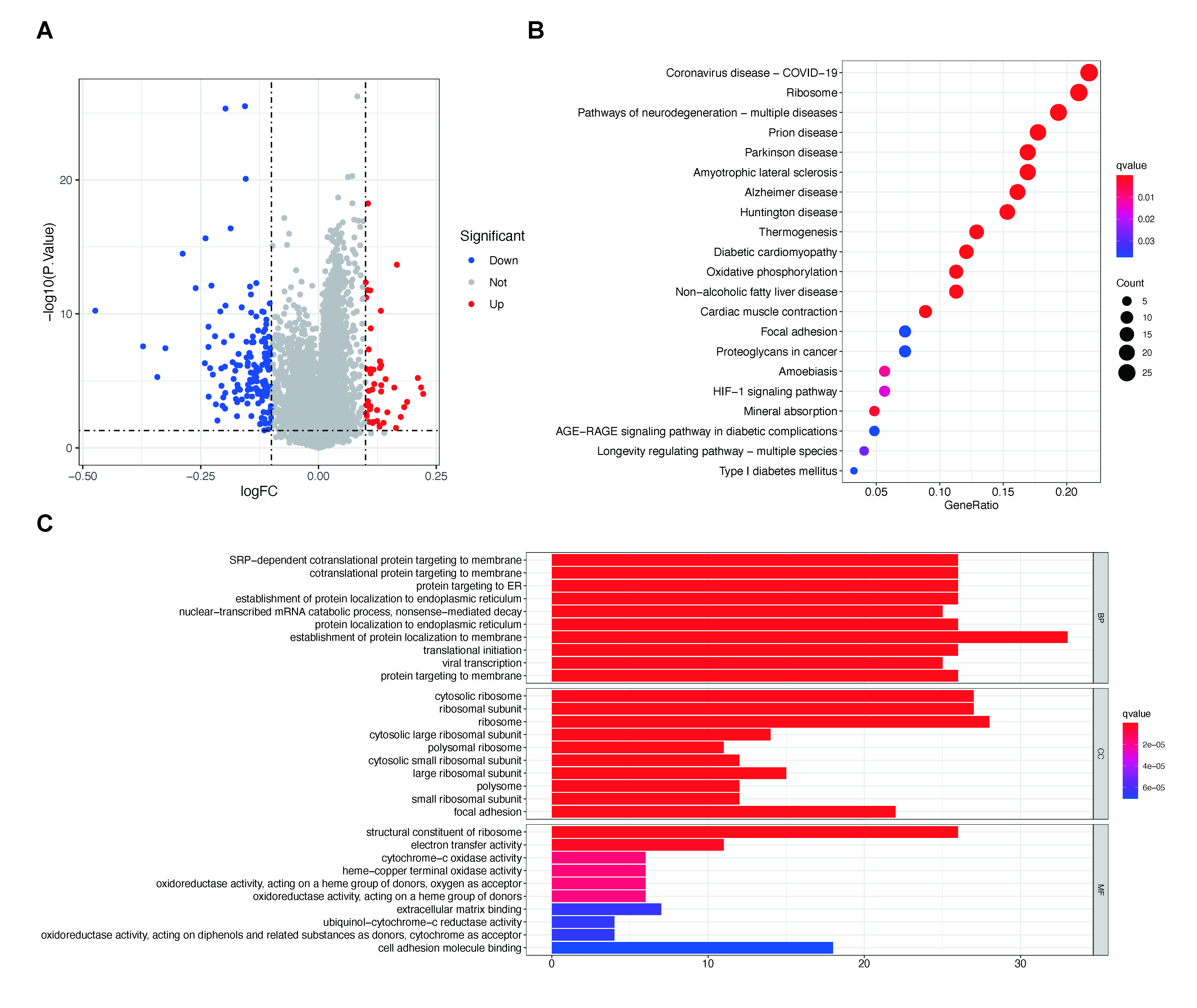

Supplement: Supplementary file 1 [file jcm-12-01341-s001.zip › Supplementary Figure S3.tif]
